# Potential role of miR-29b from mesenchymal stromal cell-derived extracellular vesicles in leukemic cell progression

**DOI:** 10.1371/journal.pone.0328922

**Published:** 2025-09-10

**Authors:** Heejei Yoon, Silvia Park, Yong-Rim Kwon, Yoo-Jin Kim

**Affiliations:** 1 Cancer Research Institute, College of Medicine, The Catholic University of Korea, Seoul, Republic of Korea; 2 Leukemia Research Institute, College of Medicine, The Catholic University of Korea, Seoul, Republic of Korea; 3 Department of Hematology, Seoul St. Mary’s Hematology Hospital, College of Medicine, The Catholic University of Korea, Seoul, Republic of Korea; European Institute of Oncology, ITALY

## Abstract

Crosstalk between leukemic cells and their surrounding mesenchymal stromal cells (MSCs) in the bone marrow microenvironment is crucial for the pathogenesis of myelodysplastic syndromes (MDS) and is mediated by extracellular vesicles (EVs). The EV-specific miRNAs derived from MDS-MSCs remain poorly explored. EVs isolated from HS-5, an immortalized stromal cell line, promoted the proliferation and 5-azacytidine (AZA) resistance of SKM-1 cells. EVs from MDS-MSCs and HS-5 cells showed significantly higher miRNA-29b-3p but lower let-7a-5p and miR-23-3p compared to healthy controls. miR-29b was selected for further investigation because it negatively regulates epigenetic modifier genes such as *DNMTs* and *TETs*, mutations of which are common in MDS. When we transduced miR-29b into leukemic cells, these cells demonstrated greater resistance to AZA and venetoclax than did their parental cells, mirroring the effect observed with HS-5 EVs. The introduction of miR-29b down-regulated *DNMT1*, *DNMT3L*, *TET1*, and *TET2*, which may underlie the changes in DNA methylation levels, increased chromosomal instability, activation of type I interferon pathways, acquired cell migration, and drug resistance observed in leukemic cells. Overall, we suggest that the sustained influx of miR-29b through EVs may catalyze the evolution of leukemic cells, and its clinical relevance warrants further investigation.

## Introduction

Myelodysplastic syndromes (MDS) are heterogeneous myeloid neoplasms with peripheral blood cytopenia due to bone marrow (BM) failure that often evolve into acute myeloid leukemia (AML) [1,2]. The overall median survival of MDS for lower- and higher-risk patients was 5.9 and 1.5 years, respectively [2]. The most common somatic mutations in MDS occur in genes affecting splicing, DNA methylation, and chromatin/histone modifications [3]. Hypomethylating agents (HMAs), such as 5-azacitidine (AZA) and 5-aza-deoxycytidine (decitabine, DEC), are commonly used to treat patients with high-risk MDS/AML, but fewer than 50% of patients respond favorably [4–6]. Treatment is limited by resistance, relapse, and a lack of biomarkers for predicting drug responses [4,7]. Therefore, new treatment strategies need to be explored to overcome these limitations.

Myeloid neoplasms such as MDS are considered a disease of the BM tissue in that reciprocal interactions between abnormal hematopoietic cells and adjacent stromal cells may underlie its initiation and progression [1]. MDS/AML cells remodel the BM microenvironment, termed the niche, and especially affect the osteogenic differentiation of mesenchymal stromal cells (MSCs) [1,8–10], which disturbs bone formation. This observation is relevant to the decrease of osteoblasts and the high prevalence of osteoporosis in patients with MDS and AML [11,12]. These MDS-derived MSCs show altered gene expression and an impaired capacity to support healthy hematopoietic stem cells (HSCs) and instead enhance the growth and survival of MDS/AML cells *ex vivo* and *in vivo* [8,13]. In contrast, an altered BM microenvironment contributes to the transformation of normal HSCs into the MDS-like phenotype. Several murine studies have suggested that the deletion of *RARγ*, *Rb*, *IkBa*, or *Sipa1* in the microenvironment and the activation of β-catenin in osteoblasts induce myeloid neoplasms in hematopoietic cells [1,14]. In particular, the deletion of *Dicer1* or *Sds* in osteoprogenitor cells predisposes normal HSCs to MDS-like phenotypes [15]. Subsequent transplantation experiments confirmed that the MDS-like phenotype was induced by the mutant BM microenvironment after transplantation of normal hematopoietic cells. As such, crosstalk between MDS/AML cells and their surrounding cells is a critical factor in the pathogenesis of MDS/AML. However, the mechanisms by which these cells communicate and lead to the development of refractory malignant tumors are not yet well understood.

Small extracellular vesicles (EVs), including exosomes, play critical roles in cell-to-cell communication [16]. MDS/AML-derived EVs containing proteins, mRNAs, lipids, and microRNAs (miRNAs) can alter the differentiation potential of MSCs in the BM microenvironment, thereby decreasing osteoblast development [8,9]. EVs derived from BM-MSCs showed differential miRNA profiles between MDS patients and healthy donors [17,18]. MSC-EVs from patients with MDS reduce MDM2 expression levels, modify the viability of HSCs [17], and induce apoptosis in normal HSCs caused by DNA damage [19]. Wang et al. demonstrated that mouse BM-MSC-derived exosomes increase the viability and proliferation of mouse myeloma cells and decrease their apoptosis in response to bortezomib [20]. These studies emphasize the important role of EVs in cell-to-cell communication, potentially explaining the phenotypic changes observed in co-culture or engraftment experiments [8,13,15]. However, the specific proteins or miRNAs within the EVs responsible for driving these phenotypes, including HMA resistance, remain poorly explored.

In the present study, we isolated EVs derived from MSCs obtained from patients with MDS (MDS-MSCs), healthy controls (HCs), and a cell line. We then identified differentially presented miRNAs within these EVs. Among them, we further investigated the effects of miRNA-29b on cell proliferation, drug responses, DNA methylation, cell migration, and chromosomal instability. Our data suggest that miR-29b may contribute to the progression of leukemic cells.

## Materials and methods

### Cell culture and reagents

HC-MSCs were purchased from the Catholic Institute of Cell Therapy (Catholic Medical Center, Seoul, Korea) and maintained in Dulbecco’s modified Eagle’s medium (DMEM; Thermo Fisher Scientific, Waltham, MA, USA) supplemented with 10% fetal bovine serum (FBS; Corning, New York, USA) and 1% penicillin/streptomycin/amphotericin B (PSA; Thermo Fisher Scientific) solution at 37 °C under a humidified atmosphere with 5% CO_2_. To establish MDS-MSCs, BM-MNCs were isolated from BM aspirates of patients with MDS using Ficoll gradient centrifugation and cultured in DMEM supplemented with 20% FBS and 1% PSA solution for several days. Cells attached to the dish substratum were collected and subsequently immunophenotyped with fluorescently labeled CD73, CD105, CD90, CD34, and CD45 antibodies (51–9007663, 51–9007661; BD Biosciences, Franklin Lakes, NJ, USA) using flow cytometry to validate MSCs. This study was approved by the Institutional Review Board of the Catholic University of Korea (KC19SES10599, KC19SESI0801) and has been performed in accordance with the Declaration of Helsinki. Written informed consent was obtained from all subjects. HS-5 cells were purchased from the American Type Culture Collection (ATCC, Manassas, VA, USA), SKM-1 from the Japanese Collection of Research Bioresources Cell Bank (JCRB Cell Bank, Osaka, Japan), and THP-1 cells from the Korean Cell Line Bank (KCLB, Seoul, Korea). HS-5 cells were cultured in DMEM supplemented with 10% FBS and 1% PSA solution. SKM-1 and THP-1 cells were cultured in RPMI-1640 (Thermo Fisher Scientific) supplemented with 10% FBS and 1% PSA solution. Cell line authentication was performed in the KCLB.

### Isolation of EVs

We performed EV isolation and characterization in compliance with the Minimal Information for Studies of Extracellular Vesicles (MISEV) guidelines [21,22]. EVs were isolated by differential centrifugation, followed by ultracentrifugation. To eliminate the exosomes from the FBS, it was centrifuged at 100,000 × g for 16 h at 4 °C. To isolate EVs from MSCs, cells at passage #3–6 were cultured to 70–80% confluence in DMEM supplemented with 10% exosome-depleted FBS and 1% PSA solution and then the medium was replaced, followed by an additional 24 h incubation. The culture media were then harvested and centrifuged at 250 × g (1200 rpm) for 10 min and at 1560 × g (3000 rpm) for 30 min to eliminate cells and cellular debris, respectively. To remove the microvesicles, the samples were centrifuged at 10,000 × g for 30 min. The pre-processed medium was further centrifuged at 110,000 × g for 90 min at 4 °C using a Sorvall WX 100 + Ultracentrifuge (Thermo Fisher Scientific). After ultracentrifugation, the pellet was resuspended in 500 µL of 1 × phosphate-buffered saline (PBS) or serum-free DMEM and stored at −80 °C for further use. The protein concentration of the exosomes was determined using Bio-Rad Protein Assay Dye Reagent Concentrate (Bio-Rad, Hercules, CA, USA) according to the manufacturer’s instructions.

### Transmission electron microscopy (TEM) for EVs

Ten microliters of EVs was loaded onto Formvar-coated nickel grids, which were then subjected to negative staining with 1% aqueous uranyl acetate. After drying, the pellets were examined using TEM (JEM-1010; Tokyo, Japan) at 100 kV as described [23].

### Nanoparticle tracking analysis (NTA) for EVs

NTAs were performed to determine the concentration and particle size of the EVs using ExoCope^TM^ mono (Exosome Plus, Inc., Korea). The samples were initially diluted in 1000 µL of filtered PBS to generate a 1 μg/mL protein concentration and further diluted if the particle concentrations did not reach the optimal range of analysis with the ExoCope program after the test. The filtered PBS was examined to ensure that it was particle-free. Each sample was injected into the cuvette using a 1 mL syringe. Measurements for the samples were repeated at least 15 times at different subvolume positions, and the replicates were averaged and are represented as a bar graph. Analyses were performed with ExoCope tracker software, version 1.005 (ExoCope monoTM).

### Western blotting

To prepare the samples, 10 µg of EVs or drug-treated cells were lysed using 5 × RIPA buffer (DyneBio, Seoul, Korea) or M-PER buffer (Thermo Fisher Scientific), both supplemented with a 1 × phosphatase and protease inhibitor cocktail (Merck Korea, Seoul, Korea). Following centrifugation, the lysates were combined with 4 × sample buffer (Bio-Rad), resolved on 4–15% or 12% precast gels (Bio-Rad), and transferred to polyvinylidene difluoride (PVDF) membranes. Membranes were blocked with 5% skim milk and then incubated with specific primary and secondary antibodies. The primary antibodies were diluted 1:2,000, while secondary antibodies were diluted between 1:5,000 and 1:10,000. Signal detection was performed using the SuperSignal West Pico Chemiluminescent Substrate (Thermo Fisher Scientific) and a Fusion SL4 image analyzer (Vilber Lourmat, France). The antibodies CD63 (EXOAB-CD63A-1; System Biosciences, Palo Alto, CA, USA), CD9 (MAB1880-SP; R&D Systems, Minneapolis, MN, USA), Calnexin (2679), RIG-I/DDX58 (3743), phospho-NF-κB p65 (Ser536) (3033), NF-κB p65 (8242), phospho-STAT1 (Tyr701) (7649), phospho-H2A.X (Ser139) (9718), PTEN (9188), phospho-AKT (Ser473) (4060), pan-AKT (4691), MCL1 (5453), BCL2 (4223), BCL-xL (2764), and ACTB (4970) were obtained from Cell Signaling Technology (Beverly, MA, USA).

### EV-specific miRNA analysis

EV RNA was isolated using TRIzol LS or the Total Exosome RNA and Protein Isolation Kit (Thermo Fisher Scientific) according to the manufacturer’s instructions. The isolated RNA concentration was determined using a NanoDrop, and its size was confirmed using an Agilent RNA 6000 Pico Kit on an Agilent 2100 Bioanalyzer (Agilent Technologies, Böblingen, Germany). miRNA profiling was performed using a TaqMan Array Card (TAC; Thermo Fisher Scientific, Inc.). We synthesized miRNA cDNA with a TaqMan Advanced miRNA cDNA synthesis kit (Thermo Fisher Scientific) and followed by quantitative real-time PCR using a TaqMan Advanced miRNA Human A Card and ViiA 7 thermocycler (Thermo Fisher Scientific). Differentially expressed miRNAs were selected based on cycle threshold (Ct) values after normalization with the limma R package (https://cran.r-project.org/).

### Small RNA-seq

We used 50 ng of EV RNA to construct a small RNA sequencing library with a SMARTer smRNA-seq kit (TaKaRa, Inc., Shiga, Japan) according to the company’s manual. Briefly, EV RNA was first polyadenylated, and followed by cDNA synthesis with PrimeScript RT and 3’ smRNA dT primers. Next, cDNA was amplified, and full-length Illumina adapters were added. The amplified libraries were analyzed on 6% PAGE gels to excise DNA bands in the range of 150–250 bp. DNA was further purified and concentrated by ethanol precipitation after overnight incubation in 0.1 × TE. The small RNA library was sequenced with a NextSeq500 (1 × 75 bp) from a local service provider (LAS, Seoul, Korea). After trimming sequencing adapters and raw quality bases [24], the reads were aligned and annotated with STAR software and miRBase. The normalization and identification of differentially expressed miRNAs were performed with DESeq2 (https://cran.r-project.org/). The differentially expressed miRNAs were visualized with volcano plots and heatmaps (https://cran.r-project.org/).

### RNA-seq analysis

Total RNA was isolated from the cell lines and MSCs tested in this study using TRIzol reagent (Thermo Fisher Scientific, Inc.) and sent to a local service provider (LAS, Seoul, Korea) for next-generation sequencing (NGS), where RNA concentration and quality were determined using a NanoDrop and an Agilent 2100 Bioanalyzer (Agilent Technologies), respectively. Paired-end sequencing libraries were constructed using an MGIEasy RNA Directional Library Prep Kit (MGI, Hong Kong, China). After sequencing, sequencing adapters and raw quality bases were trimmed by Skewer [24], and high-quality reads were then mapped to the reference genome by STAR software. The mapped reads were converted to gene expression values by Cuffquant in the Cufflinks. Genes were annotated based on reference genomes hg19 or hg38, and the expression values were calculated in fragments per kilobase of transcript per million fragments mapped (FPKM) units. The differentially expressed genes between the two selected biological conditions were identified by DESeq 2 packages. Volcano plots and heatmaps were also drawn by EnhancedVolcano and pheatmap R packages (https://cran.r-project.org/). Gene sets were analyzed by gene set enrichment analysis (GSEA) and the ClusterProfiler R package. The predicted miR-29 target genes were downloaded from TargetScan (http://targetscan.org).

### Cell proliferation assays with CCK-8 reagent

To test the effect of EVs on cell proliferation and drug resistance, cells were usually seeded at a density of 1–2 × 10^4^ cells/well in 96-well plates, and EVs were added immediately after seeding. The drugs AZA, DEC and venetoclax (VEN) were purchased from Sigma and Selleckchem (Houston, TX, USA) and added 1 d after seeding, after which the cells were cultured for 2 d. Viable cell numbers were measured using CCK-8 reagent according to the manufacturer’s protocol (Dojindo, Kumamoto, Japan). The optical density was measured at 450 and 600 nm 2–4 h after adding the CCK-8 reagent.

### Enzyme-linked immunosorbent assays (ELISAs) for IL6 and CSF2 (GM-CSF)

The culture media were removed and centrifuged at 250 × g (1200 rpm) for 10 min and at 1560 × g (3000 rpm) for 30 min to eliminate cells and cellular debris, respectively. We then performed ELISAs (ELISA MAX™ Deluxe Set) according to the manufacturer’s protocol (BioLegend, San Diego, CA, USA).

### Cell cycle and apoptosis analyses after transduction

SKM-1 or THP-1 cells were transduced with lentiviral vectors purchased from GeneCopoeia (hsa-miR-29b-1, #LPP-HmiR0120-MR03–050; scrambled control, LP502−100) according to the company’s manual. Once stabilized, transduced cells were selected using fluorescence-activated cell sorting (FACS) analysis. For cell cycle analysis, SKM-1 or THP-1 cells and miR-29b-transduced cells were washed with cold 1 × PBS and subsequently fixed with 70% cold ethanol for over 30 min at −20 °C. The cells were washed with 1 × PBS and incubated with a staining solution containing 50 μg/ml propidium iodide (PI) and 200 μg/ml RNase A for 15 min at 37 °C. DNA content was analyzed using a Becton-Dickinson FACS LSR Fortessa flow cytometer. The data were analyzed with FACSDiva software (Becton Dickinson, Heidelberg, Germany). For apoptosis analysis, drug-treated cells were stained with Annexin V-allophycocyanin (APC) and PI (BioLegend, San Diego, USA) and analyzed using flow cytometry.

### Assessment of DNA methylation with reduced representation bisulfite sequencing (RRBS)

Genomic DNA was isolated from cell lines as described above. The RRBS library was constructed using a Zymo-seq RRBS kit according to the manufacturer’s manual (Zymo Research, Irvine, CA, USA). Briefly, 500 ng of genomic DNA was digested with MspI, followed by adapter ligation and gap filling. Next, the DNA was purified and treated with bisulfite. After purification, the converted DNA was amplified with index primers and analyzed using an Agilent 2100 Bioanalyzer (Agilent Technologies). Next-generation sequencing and bioinformatics analyses were conducted by a local service provider (LAS). Paired-end sequencing (75 bp) was performed using an Illumina NextSeq 500, and sequencing adapters and raw quality bases were trimmed using Skewer [24]. The cleaned high-quality reads were mapped to the reference genome with a 3-letter-converted genome by the BS_seeker2-align module of BSseeker2 [25]. Differentially methylated cytosines (DMCs) were identified using methylKit [26]. The percentage of methylated cytosines was visualized with IGV (https://software.broadinstitute.org/software/igv/).

### Migration assay

Cells were seeded in the upper chamber of a Transwell plate (SPL, Seoul, Korea) in RPMI-1640 without FBS. In the lower chamber, the culture medium was supplemented with 10% FBS. The upper chamber was removed 24 h after seeding. The number of migrated cells was indirectly estimated using the CCK-8 reagent.

### Immunofluorescence (IF)

Cells were attached to a poly-L-lysine-coated chamber dish using centrifugation, fixed with cold methanol, permeabilized with 0.2% Triton X-100 for 10 min, and blocked with 5% FBS for 1 h at room temperature. Subsequently, the cells were incubated with APC-conjugated anti-H2A.X phospho (Ser139) antibodies (BioLegend, #613415, dilution 1:250) overnight at 4 °C. After PBS washes, the nuclei were counterstained with ProLong™ Gold Antifade Reagent (Thermo Fisher Scientific). Confocal images were captured using an LSM700 confocal microscope (Carl Zeiss A. G., Jena, Germany) and analyzed using the ZEN 3.7 program (Carl Zeiss A. G.).

### Copy number analysis and allele frequency

Genomic DNA was isolated from cell lines with a High Pure PCR Template Preparation Kit (Roche) according to the manufacturer’s instructions. The isolated DNA was digested, PCR amplified, fragmented, labeled and hybridized to a CytoScan HD Array (Affymetrix, Santa Clara, CA, USA) according to the manufacturer’s instructions. The array was then washed using Affymetrix fluidics stations and scanned using a Gene Chip Scanner 3000. The array image (CEL file) was acquired using the Affymetrix GeneChip® Operating Software (GCOS version 1.4) and further analyzed using the Chromosome Analysis Suite (ChAS version 4.2.1) software to infer the weighted log2 ratio and allele difference.

### Statistical analysis

All the data and their statistical significance were analyzed using GraphPad Prism 9 (GraphPad Software, Inc., San Diego, CA, USA). Student’s two-tailed t-test was used for statistical evaluation; **** p < 0.0001, *** p < 0.001, ** p < 0.01, and * p < 0.05 were considered statistically significant.

## Results

### EVs from HS-5 enhanced SKM-1 cell proliferation and increased cell resistance to AZA

**Fig 1:**
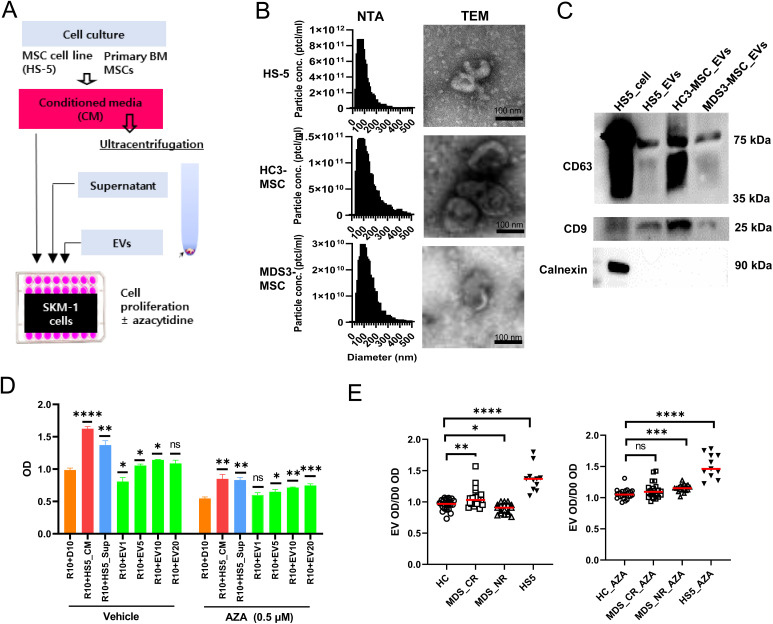
EV isolation from MSCs and their impact on SKM-1 cell proliferation and AZA resistance. **(A)** Schematic for the isolation and functional study of HS-5 cell- and primary BM-MSC-derived EVs. **(B)** The particle size, concentration, and shape of EVs were determined using a nanoparticle tracking analysis(NTA) and TEM. **(C)** Western blotting was performed for CD63 and CD9 (positive exosomal marker proteins) and Calnexin (negative exosomal marker protein). **(D)** SKM-1 cells were seeded in 96-well plates at 2 × 10^4^ cells/well in media with different compositions, including isolated EVs from HS-5 cells (HS5-EVs). Cell proliferation was assessed three days after seeding using the CCK-8 reagent. The following media compositions were tested: R10 (RPMI-1640 + 10% FBS) +D10 (DMEM + 10% FBS), R10 + HS5_CM (HS-5 cell culture-conditioned media), R10 + HS5_Sup (supernatant from ultracentrifugation for HS5-EV isolation), and R10 + EVn (DMEM + n μg/mL HS5-EVs, n = 1, 5, 10, 20). Each of these media components was mixed at a 1:1 ratio. EV concentrations were increased from 1 to 20 μg/mL. SKM-1 cells were treated with 0.5 μM AZA or vehicle one day after HS5-EV addition, and cell survival was evaluated using CCK-8 reagent after two days. An unpaired t-test was used to calculate the statistical significance against R10 + D10. **(E)** HC-MSC (normal group, HC) and MDS-MSC (MDS-CR = HMA responder and MDS-NR = HMA non-responder)-derived EVs (20 μg/mL) were incubated with SKM-1 cells for 1 day, followed by 0.5 µM AZA or vehicle exposure for two days. Cell numbers were evaluated using CCK-8 reagent. D0 represents the media composition resulting from a 1:1 mixture of R10 and D0 (DMEM + 0% FBS and other compositions as described in **(D)**). The OD values of EV-treated SKM-1 cells were normalized to the OD value at D0 in each group. The normalized values were then used to compare the differences between groups. All experiments were repeated at least three times, and representative data are presented. Statistical significance was calculated using an unpaired t-test against HC or HC_AZA: *p < 0.05, **p < 0.01, ***p < 0.001, ****p < 0.0001.

HS-5 is an immortalized MSC cell line often used as a model to study interactions between MSCs and tumor cells [27–29]. We initially optimized our methodology for EV isolation from HS-5 conditioned media (CM) and then tested the effect of CM and EVs on the proliferation of SKM-1 cells treated with or without AZA (Fig 1A). EVs were isolated by differential centrifugation, and their average size was approximately 90 nm (Fig 1B). The shape and size of these EVs were further evaluated using transmission electron microscopy (TEM) (Fig 1B), and the presence of the exosomal proteins CD9 and CD63 was confirmed with western blots (Fig 1C). HS-5-derived CM and HS-5-derived EVs (HS5-EVs) promoted SKM-1 proliferation compared to the media control, and similar results were observed after treatment with AZA, where the dose dependency of HS5-EVs was more prominent (Fig 1D). Next, we assessed EVs derived from the MSCs of both MDS patients and healthy controls. Patients with MDS were subgrouped into responders (MDS-CR, n = 5) and non-responders (MDS-NR, n = 6) based on HMA treatment (Fig 1E and S1 Table). EVs from the non-responder appeared to somewhat impair cell growth while conferring resistance to AZA (Fig 1E). Notably, the effect of the HS5-EVs on cell proliferation and AZA sensitivity was much greater than that of others (Fig 1E). These results suggested that a subset of MDS MSC-derived EVs may influence tumor cell proliferation and sensitivity to AZA.

### The NF-κB signaling pathway is activated in HS-5 cells

**Fig 2:**
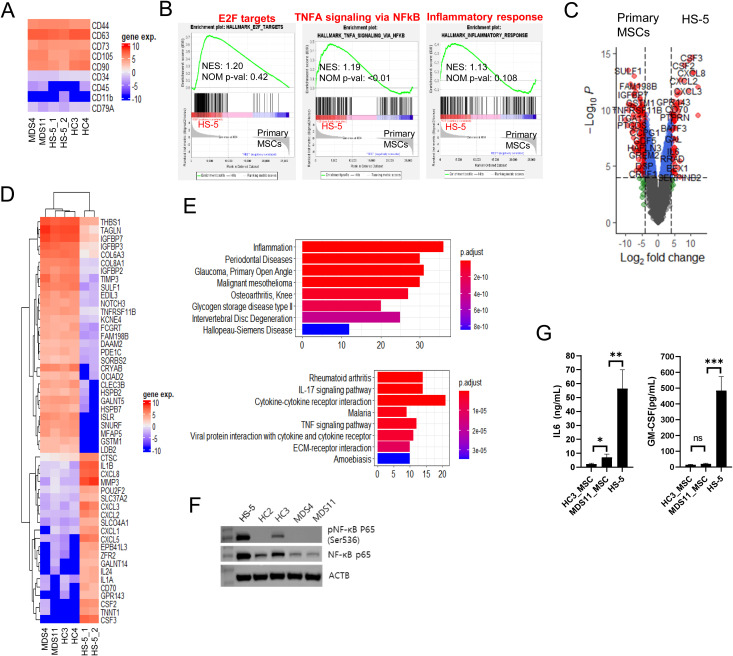
Identification of differentially expressed genes between HS-5 cells and MDS-MSCs or HC-MSCs. Total RNA was extracted from HS-5 cells, two MDS-MSCs, and two HC-MSCs (total n = 5) and subjected to bulk RNA-seq. The expression levels were quantified using FPKM values. Expression levels of MSC cell surface markers were visualized using a heatmap (A), and enriched gene sets were identified using GSEA (B). Differential expression was defined as logFC > 4 and p-value < 0.0001 and visualized using a volcano plot (C) and a heatmap (D). (E) Additional gene set analyses were performed using the DisGeNet and KEGG databases. (F) Western blotting was performed for phosphorylated NF-κB p65 (Ser536), NF-κB p65 and ACTB. (G) Secreted IL6 and CSF2 (GM-CSF) were detected using ELISAs. This experiment was repeated twice with consistent results, and representative data are shown.

HS-5 cells exhibited molecular phenotypes close to those of normal MSCs [29]. Unexpectedly, EVs derived from HS-5 substantially promoted SKM-1 cell proliferation compared to other EVs (Fig 1). To clarify this surprising outcome, we analyzed the gene expression profiles of HS-5 cells with four primary MSCs. All MSCs displayed a high degree of similarity in their surface marker expression levels (Fig 2A). Genes associated with E2F target genes, TNFA signaling via NF-κB, the inflammatory response, and rheumatoid arthritis were significantly enriched in the overexpressed genes in HS-5 cells compared to other MSCs (Fig 2B). The overexpression of E2F target genes reflects the faster growth of the HS-5 cells that we observed in cell culture. The most significantly overexpressed genes were *CSF2* (*GM-CSF*), *CSF3* (*G-CSF*), *IL1A*, *IL1B*, *IL6*, *CXCL1*, *CXCL2*, *CXCL5*, *CXCL8* (*IL8*), *MMP1*, and *MMP3* (Figs 2C and D). These genes are associated with the inflammatory response (Fig 2E). High levels of phosphorylated NF-κB p65 (Ser536), secreted IL6 and CSF2 (GM-CSF) in HS-5 cells were confirmed with western blots and ELISAs, respectively (Figs 2F and 2G). These results suggest that the NF-κB signaling pathway is strongly activated in HS-5 cells.

### miR-29b-3p was relatively abundant in MDS-MSC- and HS-5-derived EVs

Based on our above observations, we hypothesized that HS5-EVs might play a pro-tumorigenic role. Among the various cargos in EVs, we focused on the miRNAs responsible for the tumor-supportive functions.

**Fig 3:**
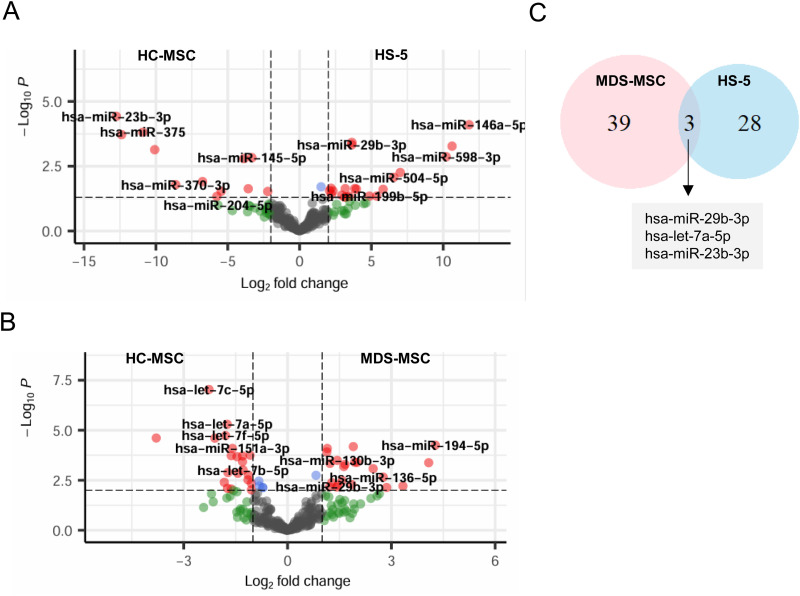
miRNAs differentially present in HS5- and MDS-EVs. Differentially expressed miRNAs (HC-MSCs vs. HS-5 or HC-MSCs vs. MDS-MSCs) were identified using qRT-PCR (A) and small RNA-seq (B). For qRT-PCR analysis, RNA was extracted from EVs derived from two HS-5 samples and HC-MSCs, and profiled using TaqMan Array Cards. Differential expression was defined as logFC > 2 and p-value < 0.05. For detailed protocols, see the Materials and Methods. For small RNA-seq, EVs from six HC-MSCs and eleven MDS-MSCs were used to construct small RNA libraries with the SMARTer smRNA-seq kit. Differential expression was determined using a cutoff of logFC > 1 and p-value < 0.01. Detailed methods are described in the Materials and Methods. Shared miRNAs are shown in the Venn diagram (C).

To identify differentially loaded miRNAs between EVs derived from HC-MSCs and those derived from HS-5 cells, we performed qRT‒PCR on an array card platform. The miR-199a-3p, miR-146a-5p, miR-29a/b-3p, and miR-369-3p levels were greater in HS5-EVs, whereas the let-7i-5p, miR-145-5p, let-7a-5p, miR-143-3p, and miR-23b-3p levels were greater in HC-MSC-EVs (Fig 3A and S2 Table). In particular, miR-146a-5p exhibited the greatest increase in expression, followed by miR-29a and 29b. miR-146a-5p is a target gene of NF-κB [30], the signaling pathway of which is activated in HS-5 cells. We also performed small RNA sequencing of 6 HC-MSC- and 11 MDS-MSC-derived EVs. Let-7 family, miR-483-3p, miR-10a/b-5p, miR-365a/b-3p, miR-151-3p, and miR-23-3p were relatively more abundant in HC-MSC-derived EVs, whereas miR-221-3p, miR-142-3p, miR-29b-3p, and miR-15a-5p were relatively more abundant in MDS-MSC-derived EVs (Fig 3B and S3 Table). Let-7a-5p, miR-23-3p, and miR-29b-3p were identified as common miRNAs in the two different comparison sample sets (Fig 3C). Notably, miR-29b-3p was more abundant in both MDS-MSC- and HS-5-derived EVs than in HC-MSC-derived EVs.

### miR-29b transduced cells enhanced cell proliferation and drug resistance

miR-29b negatively regulates epigenetic modifier genes, including *DNMTs* (*DNMT3A*/*3B* and *DNMT1* through *SP1*) and *TETs* (*TET1*, *TET2*, and *TET3*) [31–34]. Especially, loss-of-function mutations in *DNMT3A* and *TET2* are prevalent in clonal hematopoiesis [35] and myeloid neoplasms, including MDS [36]. Therefore, we hypothesized that a possible increase of miR-29b levels mediated by EV transfer might alter the inherited DNA methylation status of neoplastic cells, thereby contributing to the evolution of leukemic cells.

**Fig 4:**
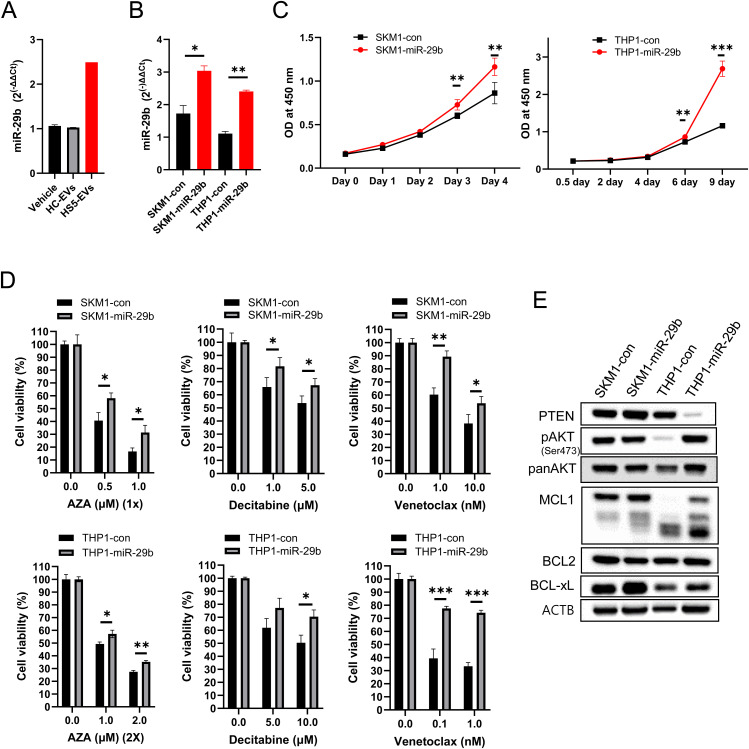
Effects of miR-29b transduction on cell proliferation and drug resistance in SKM-1 and THP-1 cells. **(A)** SKM-1 cells were incubated with HC- and HS-5-derived EVs for 24 **h.** The intracellular amount of miR-29b was determined using a TaqMan MicroRNA assay kit for miRNA-29b and RNU24. RNU24 served as a loading control. **(B)** SKM-1 and THP-1 cells were transduced with a lentiviral vector carrying either miR-29b-1 or a scrambled control. cDNA synthesis and PCR amplification were performed using the TaqMan^™^ MicroRNA Assay Kit for miRNA-29b and RNU24. **(C)** Cells were seeded at 1 × 10^4^ cells/well (SKM1-con and SKM1-miR-29b), 2 × 10^4^ cells/well (THP1-con, THP1-miR-29b), and their growth was monitored over time. CCK-8 reagent was used to evaluate cell numbers on the indicated days. **(D)** Drug responses to AZA, DEC, and VEN were assessed using the CCK-8 reagent. AZA (1×) and AZA (2×) denote single or double AZA treatments, respectively. All experiments were repeated at least three times, and representative data are presented. **(E)** Western blot was performed with the indicated antibodies.

We initially verified the increase in miR-29b expression following the addition of HS5-EVs to SKM-1 cells (Fig 4A). We then transduced SKM-1 and THP-1 cells with a lentiviral vector harboring miR-29b or a scrambled control to mimic the continuous influx of EV miR-29b into both cell lines and assessed the phenotypic changes resulting from the uptake of miR-29b. After obtaining stable transduced cells, we determined the expression level of miR-29b, which increased approximately 2-fold in the miR-29b-transduced cells (namely, SKM1-miR-29b and THP1-miR-29b) (Fig 4B); these changes stimulated cell proliferation, and the differences became evident over time in both cell lines (Fig 4C). We subsequently examined whether introducing miR-29b confers resistance to AZA, DEC, and VEN. We selected AZA, DEC, and VEN for analysis because AZA and DEC are standard treatment agents for higher-risk MDS, and recent studies have demonstrated that combination therapies incorporating VEN with these hypomethylating agents show promising efficacy not only in AML but also in higher-risk MDS. Compared with the control cells, the cells transduced with miR-29b displayed greater resistance to AZA, DEC, and VEN (Fig 4D). This resistance to AZA was further validated through DNA damage (γH2AX) and apoptosis analyses (S1A, S1B, and S1C Figs) [37–39]. VEN resistance is often associated with the up-regulation of MCL1 [40]. miR-29 down-regulates PTEN, which in turn activates the PI3K/AKT/MCL1 pathway [37–39]. These findings suggest that miR-29b may contribute to VEN resistance. Therefore, we examined the protein levels of PTEN, pAKT, MCL1, BCL-XL and BCL2 by western blots (Fig 4E). PTEN was substantially decreased in the THP1-miR-29b cells, which agreed well with the increases in phospho-AKT and MCL1. This result might explain the underlying resistance to VEN in THP1-miR-29b cells. Altogether, these results suggest that introducing miR-29b may be sufficient to phenocopy the effects of HS5-EVs on leukemic cells.

### The down-regulation of miR-29b target genes led to the activation of the interferon pathway

**Fig 5:**
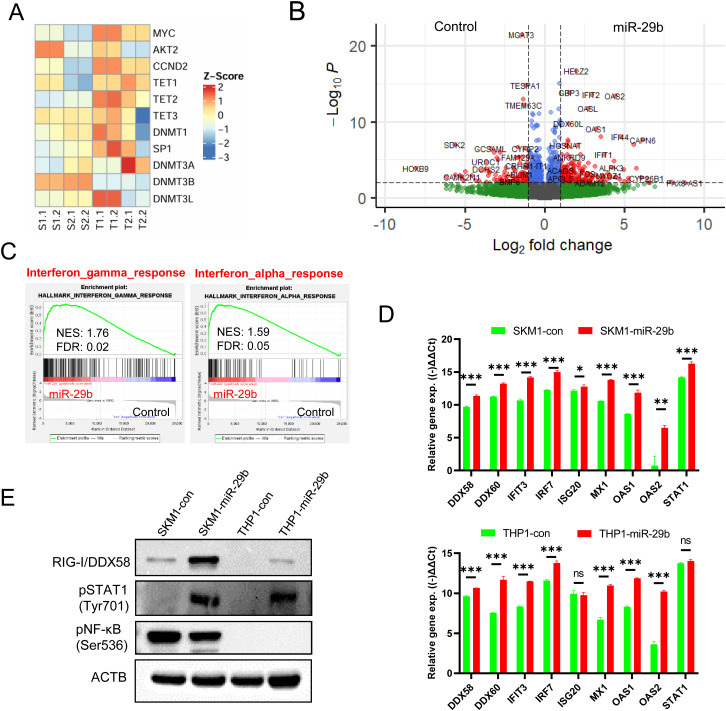
The interferon pathway is activated by miR-29b. **(A)** Expression values of miR-29 target genes were extracted from RNA-seq data, converted into Z-scores, and visualized in color code, where S1, S2, T1, and T2 represent SKM1-con, SKM1-miR-29b, THP1-con, and THP1-miR-29b, respectively. **(B)** Volcano plot displaying DEGs between the scrambled control and miR-29b-1 transduced cells. **(C)** GSEA revealed that the interferon-gamma and alpha response pathways were enriched and overexpressed in the miR-29b-1 transduced cells compared to the scrambled control cells. To confirm the activation of the interferon-gamma and alpha signaling pathways, qRT-PCR (D) and western blots (E) were conducted. Primer sequences are listed in S4 Table.

Next, we performed bulk RNA-seq to examine the down-regulation of predicted or known miR-29b target genes and to gain insights into the molecular characteristics or activated signaling pathways caused by the introduction of miR-29b. Among the predicted target genes by TargetScan (n = 753), 127 and 171 genes were down-regulated in the SKM1-miR-29b and THP1-miR-29b cells, respectively, and only 21 genes overlapped between the two (S2A Fig). Especially, the expression levels of the well-known miR-29 target genes *CCND2*, *AKT2*, and *PTEN* decreased following the introduction of miR-29b, suggesting that the introduced miR-29b functions effectively (Fig 5A and S2A Fig). The epigenetic modifier genes were not commonly down-regulated in both cell line. *TET1* expression decreased only in the SKM1-miR-29b cell line, which harbors a mutation in *TET2*, whereas *TET2* expression decreased only in the THP1-miR-29b cell line (Fig 5A and S2B Fig). No significant down-regulation were observed in the expression of *DNMT3A*/*3B*. Although not a target gene of miR-29b, *DNMT3L* was significantly down-regulated in the THP1-miR-29b cells (Fig 5A). Because DNMT3A/3B functions in combination with DNMT3L [41], this suggests that a decrease in *DNMT3L* may impair the function of DNMT3A/3B. *DNMT1* mRNA levels were decreased in the THP1-miR-29b cells but not in the SKM1-miR-29b cells.

Both miR-29b introduced cells overexpressed *IFIT2*, *OAS2*, *OASL*, and *HEL72* compared to the control cell lines (Fig 5B). Gene sets related to interferon-gamma or the alpha response were significantly enriched (Fig 5C, S2C, and S2D Figs). To confirm the activation of the pathway, qRT-PCR and western blots were performed. As shown in Fig 5D, most of the selected genes were highly expressed in the miR-29b-introduced cells, consistent with the RNA-seq results. The protein levels of RIG-I/DDX58 and phospho-STAT1 were increased in the miR-29b introduced cell lines (Fig 5E). Taken together, these data indicate that interferon pathways are activated in miR-29b introduced cell lines. However, whether the activated interferon pathways account for the observed increase in cell proliferation and drug resistance remains inconclusive.

### Changes in DNA methylation and gene expression induced by miR-29b

**Fig 6:**
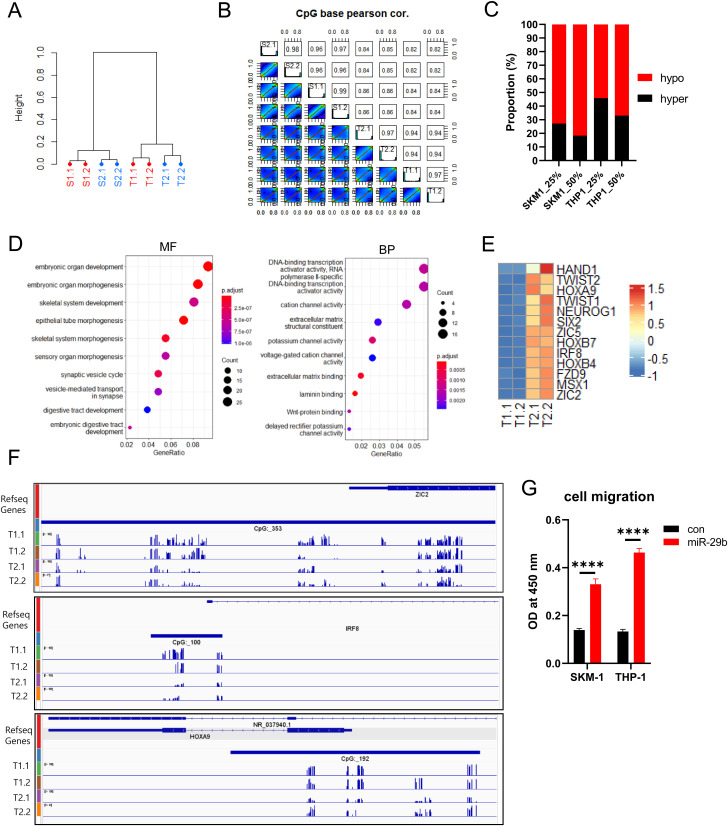
DNA methylation changes and functional effects of miR-29b overexpression. (A) and **(B)** Cluster and Pearson’s correlation analysis of DNA methylation. **(C)** The graph demonstrates the extent of hypomethylation at CpG sites with more than 25% and 50% differentially methylated cytosines in miR-29b-introduced cell lines. **(D)** Gene Ontology analysis of genes with a difference of more than 50% at one or more CpG sites near the transcription start site in THP-1 cells. MF and BP indicate molecular function and biological process, respectively. **(E)** Enrichment of genes associated with neural crest migration and HOX among hypomethylated and overexpressed genes in THP1-miR-29b cells. **(F)** Changes in DNA methylation near the TSSs of *ZIC2*, *IRF8*, and *HOXA9* were visualized using IGV. T1 and T2 indicate THP1-con and THP1-miR-29b, respectively. **(G)** Increased cell migration in THP1-miR-29b and SKM1-miR-29b cells. The experiment was repeated at least three times, and representative results are shown.

In the previous result, we observed the down-regulation of epigenetic modifier genes (*TET1*, *TET2*, *DNMT3L*, and *DNMT1*) by miR-29b in a cell-specific manner. We were curious whether these alterations lead to changes in DNA methylation levels. Therefore, we performed reduced representation bisulfite sequencing (RRBS) to identify methylation changes, primarily in CpG islands. After RRBS, the percentage of methyl C at each CpG site was determined. In the cluster analysis, cells were differentiated according to the cell line and whether miR-29b was introduced (Fig 6A). According to Pearson’s correlation analysis, the difference was about 4−6% due to the introduction of miR-29b, which was 2−3% greater than the difference in technical replication experiments within the same group (Fig 6B). More hypomethylation was observed at CpG sites showing greater than 25% and 50% of the differentially methylated cytosines in the miR-29b-introduced cell lines (Fig 6C). We further analyzed the gene ontology with genes showing differences of more than 50% at one or more CpG sites near the transcription start site in THP-1 cells. We observed that the transcription factors involved in organogenesis were highly enriched (Fig 6D). Among the hypomethylated genes with increased gene expression, genes associated with neural crest migration, such as *HAND1*, *TWIST1/2*, *ZIC2*, and *ZIC5,* were notably enriched (Fig 6E). *HOX* genes are also enriched, and *HOXA9* has been reported to be associated with cell proliferation and poor prognosis in AML [42,43]. We further verified the existence of a cluster of hypomethylated Cs near the transcription start site through visualization of methylation changes (Fig 6F). To investigate whether the up-regulation of neural crest migration genes affects the migration of THP-1 cells, we tested cell migration using a Transwell assay. Many THP1-miR-29b cells showed increased migration at different FBS concentrations (Fig 6G). We also observed increased migration of SKM1-miR-29b cells (Fig 6G). Taken together, these results confirm that the introduction of miR-29b induces changes in DNA methylation, which may lead to further changes in the cell phenotype.

### miR-29b increased chromosomal instability

**Fig 7:**
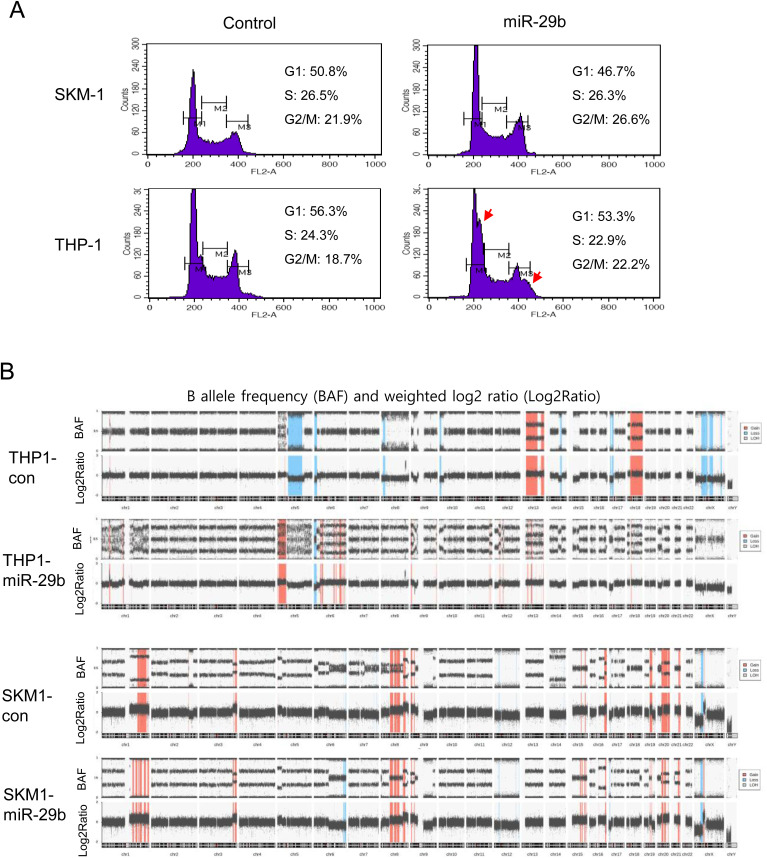
Impact of miR-29b on the cell cycle and chromosomal stability in cancer cell lines. **(A)** SKM1-con and THP1-con cells, along with miR-29b-transduced cells, were fixed with cold ethanol, stained with propidium iodide (PI) and RNase A, and analyzed for DNA content using a flow cytometer. The data were analyzed using FACSDiva software. The experiment was repeated at least three times, and representative data are shown. **(B)** The isolated genomic DNA was processed, labeled, and hybridized to a CytoScan HD array. Data analyses were performed using ChAS software to determine the weighted log2 ratio and allele difference.

Because the miR-29b introduced cell lines grew faster than the control cell lines (Fig 4C), we assumed that there was a difference in the cell cycle. Therefore, we analyzed cell cycle changes using FACS, which is based on changes in the amount of DNA. There was no significant difference in the number of cells in the S phase, whereas a greater percentage of the miR-29b-introduced cells tended to accumulate in the G2/M phase (Fig 7A). Interestingly, in the THP-1 cells, additional peaks with increased DNA amount were clearly seen in the G1 and G2/M phases in the miR-29b-introduced cells, as indicated by the arrows (Fig 7A). This finding indicated that some of the miR-29b introduced cells contained more DNA in the G1 and G2/M phases. This finding suggested that miR-29b might increase chromosomal instability or cause defects in chromosome segregation. To confirm this, we analyzed the copy number and allele frequency using a SNP array. Fig 7B shows the difference in the B-allele frequency (BAF) plot and weighted log2 ratio between the two compared cell lines. Theoretically, a single chromosome is displayed in two lines on the BAF track, two chromosomes (normal copy) in three lines, and three chromosomes in four lines [44]. In the THP1-con cells, 3 lines appeared predominantly in the entire chromosome, whereas chromosomal regions with 5 or more lines were predominantly distributed in the THP1-miR-29b cells. Similar results were not observed in SKM-1 cell lines; however, traces of partial chromosomal deletions and duplications were identified. In conclusion, the introduction of miR-29b into cancer cell lines resulted in increased chromosomal instability, leading to additional chromosomal aberrations.

## Discussion

The present study was designed to explore the role of extracellular vesicles (EVs) derived from bone marrow stromal cells in the pathogenesis and treatment resistance of myelodysplastic syndromes (MDS). Increasing evidence suggests that EV-mediated crosstalk between leukemic cells and mesenchymal stromal cells (MSCs) contributes to disease progression by transferring bioactive molecules. We focused on EV-associated microRNAs and identified miR-29b as a candidate mediator implicated in epigenetic dysregulation, chromosomal instability, and drug resistance. HS-5 cells, an immortalized BM-derived stromal cell line established via HPV E6 and E7 gene transduction, previously shown to share partial molecular similarities with healthy control MSCs [28,29]. We observed that HS-5-derived EVs exerted a more pronounced effect on leukemic cell proliferation and azacitidine (AZA) resistance compared to EVs from HC- or MDS-MSCs. Gene expression profiling revealed marked upregulation of NF-κB-driven inflammatory genes, including IL6 and CSF2 (GM-CSF), in HS-5 cells, consistent with earlier findings that immortalization may endow stromal cells with pro-tumorigenic properties [28]. These inflammatory features likely contribute to the enhanced leukemogenic potential of HS-5-derived EVs.

MicroRNAs (miRNAs) are small non-coding RNAs that post-transcriptionally down-regulate their target mRNAs. miR-29 consists of three members, namely, miR-29a, miR-29b, and miR-29c [45], and is known to regulate multiple biological processes, such as proliferation, apoptosis, metastasis, fibrosis, metabolism, angiogenesis, and epigenetics [46]. In cancer studies, miR-29 was identified as a tumor suppressor in most cases, including in AML [45]. Only a few studies have reported the pro-tumorigenic role of miR-29 [45,47,48]. Of note, lower expression levels of miR-29 were associated with favorable outcomes in patients with CLL [49]. Interestingly, miR-29 negatively regulates epigenetic modifiers such as *DNMTs* and *TETs* [31–34], which are essential for normal hematopoiesis and are most commonly mutated in MDS/AML [36]. A very poor overall survival tendency was reported in a murine model that exhibited additional *TET* or *DNMT* loss compared to single *TET* or *DNMT* loss [50,51]. This finding suggested that the influx of miR-29 into MDS cells may further increase epigenetic instability by targeting additional *TETs* and/or *DNMTs*.

Chen et al. demonstrated that the forced expression of *TET*-targeting miR-29 disrupts normal hematopoiesis and induces myeloid-biased hematopoietic expansion in a murine model [34]. Especially, miR-29a is essential for HSC self-renewal [47], and its ectopic expression can transform mouse HSCs/progenitors into leukemia stem cells [48], which are responsible for drug resistance and the development of refractory or relapsed AML [52]. Regarding HMA treatment and miR-29 expression, responders to decitabine showed higher levels of miR-29b than non-responders did [53]. In contrast, lower expression of miR-29c at diagnosis is associated with prolonged survival after 5-AZA treatment [54]. However, the mechanisms underlying these conflicting results remain unknown. These findings encouraged us to test whether the stable expression of miR-29b in MDS/AML cells, mimicking sustained transfer through EVs, might confer outgrowth and drug resistance (HMA) through epigenetic reprogramming.

We observed DNA methylation changes in CpG islands and increased chromosomal instability after the introduction of miR-29b into SKM-1 and THP-1 cells. In THP1-miR-29b, decreased expression of *TET2*, *DNMT1*, and *DNMT3L* may alter the inherited DNA methylation pattern of the parental cells, which underlies the strong chromosomal instability of THP1-miR-29b. Deficiency in *DNMTs* or *TETs* promotes chromosomal instability through DNA hypomethylation, particularly in repetitive elements and centromeric or subtelomeric sequences [55–60]. In particular, DNMT3B/Dnmt3b is specifically required for the methylation of centromeric minor satellite repeats, and defects in DNMT3B/Dnmt3b result in chromosome missegregation [55,61]. Deletion of a single *TET* gene increases DNA damage [62], and deletion of all three *TET* genes causes severe chromosomal instability [59], which is related to hypomethylation of heterochromatin regions. Although *DNMT3L* is not a predicted target gene of miR-29, its down-regulation by miR-29 has also been reported in mouse experiments [48], in agreement with our result, suggesting that miR-29b might indirectly regulate *DNMT3L*. As *TP53* mutations contribute to an increase in the degree of chromosomal instability [63], we cannot exclude the potential involvement of biallelic *TP53* mutations already present in SKM-1 and THP-1 cells in further exacerbating chromosomal instability. Our results suggest that the introduction of extrinsic miR-29b can contribute to leukemia heterogeneity and progression through epigenetic and chromosomal instability.

The introduction of miR-29b conferred modest resistance to AZA and DEC, which supports the observation that EVs derived from HS-5 and non-responder MSCs exhibited resistance to AZA. However, the involvement of other miRNAs and cytokines cannot be excluded. Although the anti-apoptotic gene MLC1 is known to be a target of miR-29b [64], miR-29b did not decrease MCL1 in SKM1-miR-29b cells. Instead, it increased MCL1 expression through PTEN down-regulation in THP1-miR-29b cells, which may confer VEN resistance. miR-29b conferred an anti-apoptotic property to AZA. It is possible that the increase in BCL-xL or MCL1 may be responsible for this effect, but the underlying mechanism has not yet been identified.

In conclusion, we identified miRNA-29b-3p in EVs derived from HS-5 and MDS-MSCs and studied its effect on leukemic cells. Introducing miR-29b down-regulated *DNMTs* and/or *TETs*, leading to changes in DNA methylation, chromosomal instability, and drug sensitivity. Our findings underscore the potential impact of EV-mediated miRNA transfer in driving MDS/AML heterogeneity and progression.

## Supporting information

S1 Fig miR-29 overexpressing cells exhibited increased resistance to apoptosis. (A) and (B) After AZA treatment at the indicated doses, apoptosis was analyzed using an annexin V and PI staining kit. (C) SKM1-con and SKM1-miR-29b cells were treated with 0.5 µM AZA for 24 h, and IF was conducted with an anti-γH2AX antibody to detect double-strand breaks.
(TIF)

S2 Fig Identification of down-regulated genes among the predicted target genes of miR-29 and the list of genes associated with the interferon-gamma response. (A) Bulk RNA-seq analysis was conducted, and down-regulated genes in miR-29b introduced cells were identified among the predicted target genes of miR-29 according to TargetScan. Only 21 genes (8%) were commonly down-regulated in both SKM1-miR-29b and THP1-miR-29b cells. (B) To validate the RNA-seq results, qRT-PCR was performed for *DNMT1*, *DNMT3A*, *TET1*, and *TET2*. (C) The genes associated with the interferon-gamma response and those up-regulated in the miR-29 introduced cells (S2 and T2) were compared to those in the parental cells (S1 and T1). (D) Gene set analyses were performed using the KEGG database.
(TIF)

S1 Table Bone marrow MSCs were isolated from MDS patients. **MDS patients were classified according to the HMA response and subtypes as previously described [65].** Abbreviations: MDS-EB2, Myelodysplastic syndrome (MDS) with Excess Blasts-2; MDS-EB1, MDS with Excess Blasts-1; MDS-MLD, MDS with multilineage dysplasia; CMML-2, Chronic Myelomonocytoc leukemia-2; IPSS-R, Revised International Prognostic Scoring System; HMA, hypomethylating agent; AZA, azacitidine; DEC, decitabine; CR, complete remission; MCR-HI, marrow CR with hematologic improvement (HI); SD + HI, stable disease with hematologic improvement; SD-HI, stable disease withouth hematologic improvement; DP, disease progression; NA, not applicable.
(DOC)

S2 Table EV-specific miRNAs differentially loaded between HS-5 cells and normal MSCs. (DOC)

S3 Table EV-specific miRNAs differentially loaded between HC-MSCs and MDS-MSCs. (DOC)

S4 Table Primer sets for RT-PCR. (DOC)
