# Supplementary figures and images for: Potential role of miR-29b from mesenchymal stromal cell-derived extracellular vesicles in leukemic cell progression
Source: PLoS One. 2025 Sep 10;20(9):e0328922. doi: 10.1371/journal.pone.0328922 (PMC12422469; doi:10.1371/journal.pone.0328922)

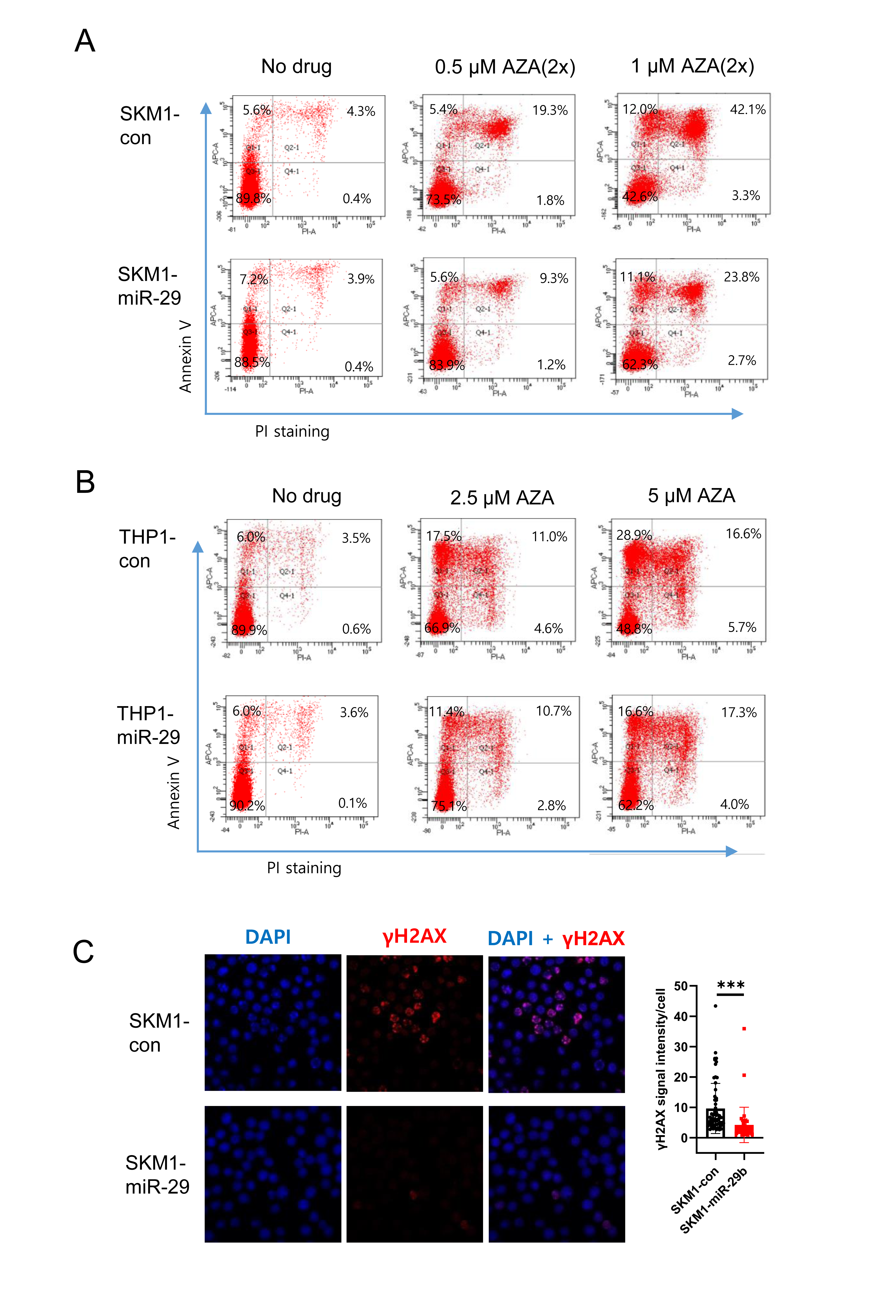

Supplement: S1 Fig — (A) and (B) After AZA treatment at the indicated doses, apoptosis was analyzed using an annexin V and PI staining kit. (C) SKM1-con and SKM1-miR-29b cells were treated with 0.5 µM AZA for 24 h, and IF was conducted with an anti-γH2AX antibody to detect double-strand breaks. (TIF) [file pone.0328922.s001.tif]

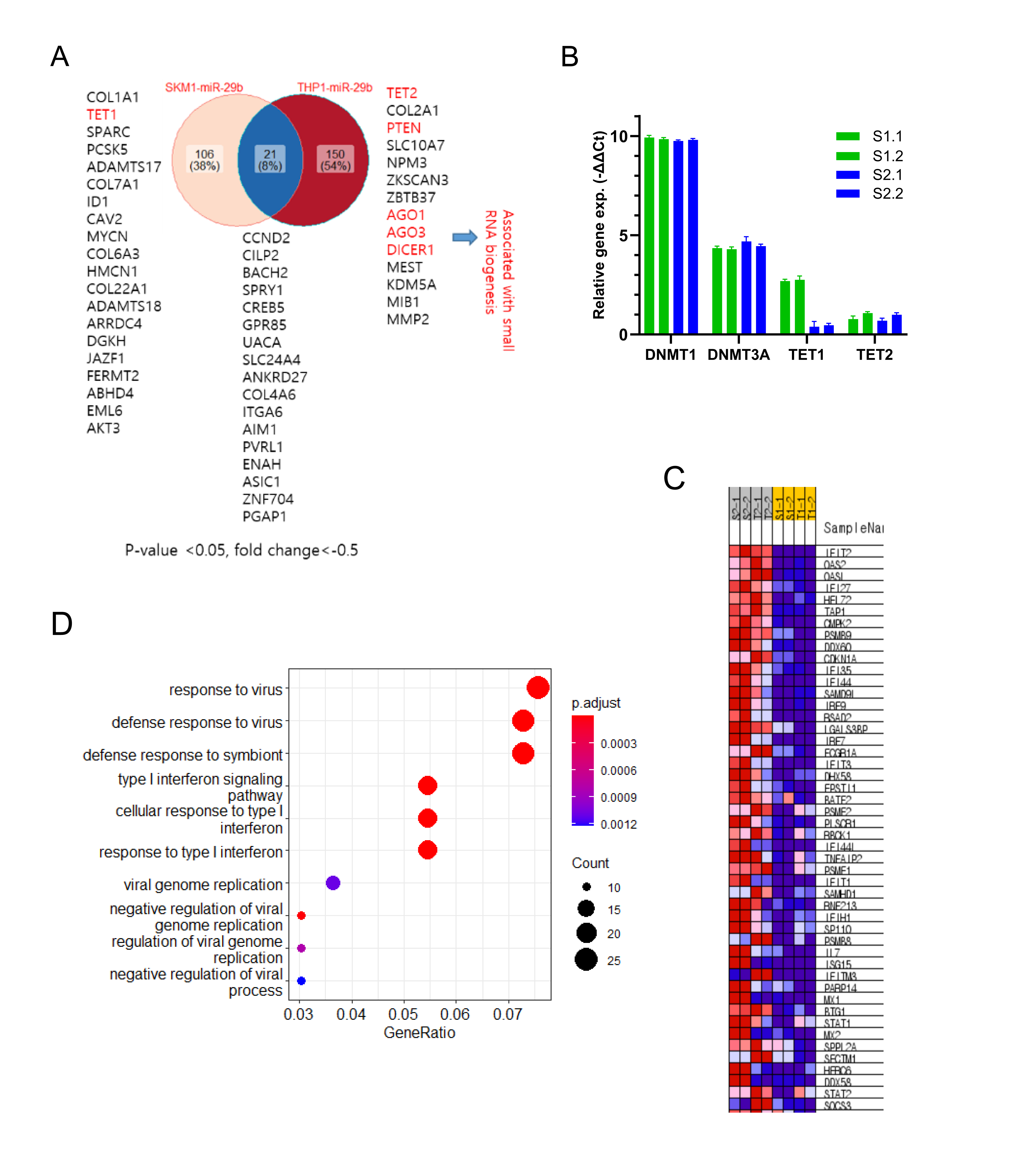

Supplement: S2 Fig — (A) Bulk RNA-seq analysis was conducted, and down-regulated genes in miR-29b introduced cells were identified among the predicted target genes of miR-29 according to TargetScan. Only 21 genes (8%) were commonly down-regulated in both SKM1-miR-29b and THP1-miR-29b cells. (B) To validate the RNA-seq results, qRT-PCR was performed for DNMT1, DNMT3A, TET1, and TET2. (C) The genes associated with the interferon-gamma response and those up-regulated in the miR-29 introduced cells (S2 and T2) were compared to those in the parental cells (S1 and T1). (D) Gene set analyses were performed using the KEGG database. (TIF) [file pone.0328922.s002.tif]
