# Supplementary material for: Potential role of miR-29b from mesenchymal stromal cell-derived extracellular vesicles in leukemic cell progression
Source: PLoS One. 2025 Sep 10;20(9):e0328922. doi: 10.1371/journal.pone.0328922 (PMC12422469; doi:10.1371/journal.pone.0328922)
Supplement: S1 Table — MDS patients were classified according to the HMA response and subtypes as previously described [65]. Abbreviations: MDS-EB2, Myelodysplastic syndrome (MDS) with Excess Blasts-2; MDS-EB1, MDS with Excess Blasts-1; MDS-MLD, MDS with multilineage dysplasia; CMML-2, Chronic Myelomonocytoc leukemia-2; IPSS-R, Revised International Prognostic Scoring System; HMA, hypomethylating agent; AZA, azacitidine; DEC, decitabine; CR, complete remission; MCR-HI, marrow CR with hematologic improvement (HI); SD + HI, stable disease with hematologic improvement; SD-HI, stable disease withouth hematologic improvement; DP, disease progression; NA, not applicable. (DOC) [file pone.0328922.s003.doc]

**S1 Table. Bone marrow MSCs were isolated from MDS patients. MDS patients were classified according to the HMA response and subtypes as previously described (65)**

| **Case_No.** | **Sex/Age** | **WHO 2016** | **BM blast (%)** | **Karyotype** | **IPSS-R score (group)** | **HMA** | **Response** |
| --- | --- | --- | --- | --- | --- | --- | --- |
| MDS1 | M/63 | MDS-EB2 | 10 | 46,XY[20] | 5.5 (High) | AZA | CR |
| MDS2 | M/67 | MDS-EB2 | 10 | 46,XY[20] | 3.5 (INT) | AZA | CR |
| MDS3 | M/60 | MDS-EB1 | 9 | 45~46,XY,add(5)(q13),-7,del(9)(q13),del(12)(p11.2),+17[cp25]/39~42,X,-Y,-5,-7,del(9)(q13),-16,-19,add(22)(q13),+add(22)(q13),+mar[cp9]/66~83,XY,+1,+2,+2, +3,+4,+4,+5,+6,+8,+8,+10,+10,+11,+12,+13,+14,+add(14)(q32),+15,+add(15)(q26.1),+17,+20,+21,+21,+add(22)(q13),+add(22)(q13)[cp5]/46,XY[1] | 8.5 (Very high) | DEC | MCR-HI |
| MDS4 | M/63 | MDS-MLD | 3 | 45,X,-Y[5]/46,XY[15] | 3.5 (INT) | AZA | SD+HI |
| MDS5 | M/52 | MDS-EB2 | 12 | 46,XY[10] | 5 (High) | AZA | SD+HI |
| MDS6 | M/51 | CMML-2 | 16 | 46,XY[20] | 5 (High) | DEC | No response  (1 failure with SD-HI) |
| MDS7 | F/37 | MDS-EB2 | 10 | 46,XX,del(20)(q11.2q13.1)[7]/46,idem,del(12)(p11.2p12)[9]/46,XX[4] | 5.5 (High) | AZA | No response  (1 failure with DP) |
| MDS8 | M/55 | MDS-MLD | 0.5 | 46,XY[20] | 3 (Low) | AZA | No response  (1 failure with SD-HI) |
| MDS9 | M/50 | MDS-EB2 | 11 | 47,XY,+add(1)(p13)[22]/46,idem,-Y[4]/46,XY,dup(1)(q21q32)[2]/46,XY[2] | 7 (Very High) | DEC | No response  (1 failure with SD-HI) |
| MDS10 | M/66 | MDS-EB1 | 9 | 46,XY,t(11;19)(q23;p13.1)[14]/45,idem,dic(11;17)(p15;p13)[1]/46,XY[5] | 6.5 (Very High) | AZA | No response  (1 failure with DP) |
| MDS11 | M/64 | MDS-MLD | 3 | 46,XY,del(1)(p34.3),?der(5)t(5;7)(q13;p13),-7,+8[20] | 7 (Very High) | AZA | No response  (1 failure with SD-HI) |
