# Supplementary material for: Potential role of miR-29b from mesenchymal stromal cell-derived extracellular vesicles in leukemic cell progression
Source: PLoS One. 2025 Sep 10;20(9):e0328922. doi: 10.1371/journal.pone.0328922 (PMC12422469; doi:10.1371/journal.pone.0328922)
Supplement: S2 Table — (DOC) [file pone.0328922.s004.doc]

**S2 Table. EV-specific miRNAs differentially loaded between HS-5 cells and normal MSCs.**

| microRNA | Ct value | | | | logFC (HS5/HC) | p.value | adj.P.Val |
| --- | --- | --- | --- | --- | --- | --- | --- |
|  | HS5_1 | HS5_2 | HC3-EVs | HC4-EVs |  |  |  |
| hsa-miR-146a-5p | 23.746 | 20.388 | 32.578 | 34.451 | 11.80 | <0.001 | 0.008 |
| hsa-miR-32-5p | 32.604 | 28.441 | 40 | 40 | 10.61 | 0.001 | 0.016 |
| hsa-miR-598-3p | 33.713 | 28.167 | 40 | 40 | 10.20 | 0.001 | 0.031 |
| hsa-miR-548am-5p_hsa-miR-548c-5p_hsa-miR-548o-5p | 34.623 | 33.461 | 40 | 40 | 7.01 | 0.006 | 0.098 |
| hsa-miR-504-5p | 34.758 | 34.236 | 40 | 40 | 6.55 | 0.009 | 0.140 |
| hsa-miR-381-3p | 28.288 | 27.69 | 32.456 | 30.83 | 3.84 | 0.023 | 0.240 |
| hsa-miR-363-3p | 28.823 | 26.423 | 29.59 | 32.823 | 3.96 | 0.024 | 0.240 |
| hsa-miR-29a-3p | 22.001 | 20.94 | 24.693 | 25.058 | 3.64 | <0.001 | 0.016 |
| hsa-miR-29b-3p | 22.783 | 21.022 | 25.308 | 25.257 | 3.63 | <0.001 | 0.016 |
| hsa-miR-296-5p | 29.655 | 27.411 | 30.086 | 33.266 | 3.48 | 0.046 | 0.309 |
| hsa-miR-15b-5p | 27.312 | 23.892 | 27.625 | 29.15 | 3.18 | 0.023 | 0.240 |
| hsa-miR-19b-3p | 27.172 | 23.305 | 28.735 | 27.031 | 2.94 | 0.042 | 0.309 |
| hsa-miR-455-3p | 25.48 | 24.73 | 27.595 | 26.734 | 2.21 | 0.039 | 0.308 |
| hsa-miR-155-5p | 26.34 | 24.433 | 27.167 | 27.684 | 2.33 | 0.028 | 0.246 |
| hsa-miR-424-5p | 25.189 | 23.833 | 26.664 | 26.114 | 2.09 | 0.028 | 0.246 |
| hsa-miR-369-3p | 25.063 | 22.861 | 25.146 | 26.5 | 2.19 | 0.022 | 0.240 |
| hsa-miR-199a-3p_hsa-miR-199b-3p | 20.911 | 19.693 | 21.768 | 21.396 | 1.50 | 0.02 | 0.240 |
| hsa-let-7a-5p | 25.893 | 26.037 | 23.153 | 23.977 | -2.21 | 0.03 | 0.246 |
| hsa-miR-145-5p | 25.263 | 23.812 | 20.203 | 21.652 | -3.31 | 0.001 | 0.031 |
| hsa-let-7i-5p | 25.113 | 25.182 | 23.089 | 19.978 | -3.57 | 0.023 | 0.240 |
| hsa-miR-143-3p | 28.212 | 25.871 | 22.581 | 23.148 | -3.87 | 0.002 | 0.031 |
| hsa-miR-217 | 40 | 40 | 35.068 | 31.546 | -6.75 | 0.012 | 0.191 |
| hsa-miR-370-3p | 40 | 40 | 27.338 | 34.893 | -8.62 | 0.016 | 0.232 |
| hsa-miR-187-3p | 40 | 40 | 31.233 | 28.743 | -10.08 | 0.001 | 0.019 |
| hsa-miR-375 | 40 | 40 | 28.976 | 29.15 | -10.91 | <0.001 | 0.010 |
| hsa-miR-412-3p | 40 | 40 | 28.951 | 26.486 | -12.39 | <0.001 | 0.010 |
| hsa-miR-23b-3p | 40 | 40 | 27.044 | 27.38 | -12.75 | <0.001 | 0.008 |
