# Supplementary material for: Potential role of miR-29b from mesenchymal stromal cell-derived extracellular vesicles in leukemic cell progression
Source: PLoS One. 2025 Sep 10;20(9):e0328922. doi: 10.1371/journal.pone.0328922 (PMC12422469; doi:10.1371/journal.pone.0328922)
Supplement: S3 Table — (DOC) [file pone.0328922.s005.doc]

**S3 Table. EV-specific miRNAs differentially loaded between HC-MSCs and MDS-MSCs**

| microRNA | log2Fold Change | p-value | adjust p-value |  |
| --- | --- | --- | --- | --- |
| hsa-miR-483-3p | -3.79 | <0.001 | 0.002 | High in HC |
| hsa-let-7c-5p | -2.26 | <0.001 | <0.001 |  |
| hsa-miR-10b-5p | -2.09 | <0.001 | 0.002 |  |
| hsa-miR-10a-5p | -1.81 | 0.004 | 0.04 |  |
| hsa-let-7f-5p | -1.78 | <0.001 | 0.001 |  |
| hsa-let-7a-5p | -1.73 | <0.001 | <0.001 |  |
| hsa-miR-365a-3p | -1.72 | 0.007 | 0.05 |  |
| hsa-miR-374b-5p | -1.71 | 0.001 | 0.02 |  |
| hsa-miR-365b-3p | -1.61 | 0.008 | 0.06 |  |
| hsa-miR-196a-5p | -1.61 | <0.001 | 0.004 |  |
| hsa-miR-151a-3p | -1.58 | <0.001 | 0.003 |  |
| hsa-miR-196b-5p | -1.45 | <0.001 | 0.005 |  |
| hsa-let-7b-3p | -1.42 | 0.001 | 0.015 |  |
| hsa-miR-424-3p | -1.3 | <0.001 | 0.004 |  |
| hsa-miR-126-5p | -1.3 | <0.001 | 0.006 |  |
| hsa-let-7b-5p | -1.27 | <0.001 | 0.011 |  |
| hsa-miR-21-5p | -1.13 | 0.003 | 0.028 |  |
| hsa-miR-574-3p | -1.12 | 0.001 | 0.017 |  |
| hsa-miR-23b-3p | -1.08 | <0.001 | 0.004 |  |
| hsa-miR-193b-5p | -1.05 | 0.01 | 0.062 |  |
| hsa-let-7e-5p | -1.03 | 0.004 | 0.037 |  |
| hsa-miR-194-5p | 4.26 | <0.001 | 0.003 | High in MDS |
| hsa-miR-629-5p | 4.07 | <0.001 | 0.007 |  |
| hsa-miR-15a-5p | 3.33 | 0.009 | 0.059 |  |
| hsa-miR-455-5p | 2.87 | 0.009 | 0.06 |  |
| hsa-miR-136-5p | 2.77 | 0.002 | 0.024 |  |
| hsa-miR-29a-5p | 2.49 | <0.001 | 0.011 |  |
| hsa-miR-144-3p | 1.99 | <0.001 | 0.007 |  |
| hsa-miR-138-5p | 1.94 | <0.001 | 0.006 |  |
| hsa-miR-1307-3p | 1.89 | <0.001 | 0.003 |  |
| hsa-miR-106a-5p | 1.85 | 0.006 | 0.046 |  |
| hsa-miR-142-3p | 1.65 | <0.001 | 0.007 |  |
| hsa-miR-192-5p | 1.61 | <0.001 | 0.01 |  |
| hsa-miR-377-3p | 1.58 | 0.003 | 0.032 |  |
| hsa-miR-432-5p | 1.45 | 0.006 | 0.046 |  |
| hsa-miR-130b-3p | 1.44 | <0.001 | 0.006 |  |
| hsa-miR-378a-3p | 1.44 | 0.007 | 0.049 |  |
| hsa-miR-301a-3p | 1.28 | 0.004 | 0.037 |  |
| hsa-miR-29b-3p | 1.22 | 0.005 | 0.038 |  |
| hsa-miR-27a-3p | 1.22 | <0.001 | 0.007 |  |
| hsa-miR-125b-5p | 1.14 | <0.001 | 0.003 |  |
| hsa-miR-221-3p | 1.13 | <0.001 | 0.004 |  |
