# Supplementary material for: Potential role of miR-29b from mesenchymal stromal cell-derived extracellular vesicles in leukemic cell progression
Source: PLoS One. 2025 Sep 10;20(9):e0328922. doi: 10.1371/journal.pone.0328922 (PMC12422469; doi:10.1371/journal.pone.0328922)
Supplement: S4 Table — (DOC) [file pone.0328922.s006.doc]

**S4 Table**. Primer sets for RT-PCR.

| Primer name | Sequence |
| --- | --- |
| DDX60 | Fw: AGAAACTCCAAACCATGTTACTC (23 mer)  Rev: TCTCCTTCATTCATCCTGTTATC (23 mer) |
| DDX58 | Fw: ACGTAAGAGTGATAGAGGAATG (23 mer)  Rev: TGTCTTGTACTTCACATGGATTC (23 mer) |
| OAS-1 | Fw: CGAAACCCAGGCCTGTGATC (20 mer)  Rev: CCCATCCCAATTCTTAAAGCATG (23 mer) |
| OAS-2 | Fw: AAGCACTGGTACAAAGAGTGTG (22 mer)  Rev: TGACCAGCTCCAGGACTGTC (20 mer) |
| MX1 | Fw: CTGATGGCCTATCACCAGGAG (21 mer)  Rev: GGAACTTCCGCTTGTCGCTG (20 mer) |
| IFIT3 | Fw: GCAGTCATGAGTGAGGTCAC (20 mer)  Rev: GCTGCCTCGTTGTTACCATC (20 mer) |
| IRF7 | Fw: TGGTCCTGGTGAAGCTGGAAC (21 mer)  Rev: GTTCTCATTAGACTGGGTTCTAG (23 mer) |
| ISG20 | Fw: TACACAAGAGCATCCAGAACAG (22 mer)  Rev: CAAAAGCAAGGTAGTTGCTGTC (22 mer) |
| STAT1 | Fw: TCTGTAGAATTCGACAGTATGATG (24 mer)  Rev: ACTTGCTATCAACAGGTTGCAG (22 mer) |
